# Awareness of Universal Design for Learning among anatomy educators in higher level institutions in the Republic of Ireland and United Kingdom

**DOI:** 10.1002/ca.23947

**Published:** 2022-09-20

**Authors:** Audrey M. K. Dempsey, Eithne Hunt, Mutahira Lone, Yvonne M. Nolan

**Affiliations:** ^1^ Department of Anatomy and Neuroscience, School of Medicine University College Cork Cork Ireland; ^2^ Department of Occupational Science and Occupational Therapy, School of Clinical Therapies University College Cork Cork Ireland

**Keywords:** anatomy, curriculum design, universal design for learning

## Abstract

There is an increasing need to facilitate enhanced student engagement in anatomy education. Higher education students differ in academic preferences and abilities and so, not all teaching strategies suit all students. Therefore, it is suggested that curricula design and delivery adapt to sustain learner engagement. Enhanced learner engagement is a fundamental feature of Universal Design for Learning (UDL). The aim of this study is to determine if anatomy educators in the Republic of Ireland (ROI) and United Kingdom (UK) are aware of UDL and to assess if, and to what extent, it has been implemented in the design and delivery of anatomy curricula for healthcare students. An anonymous online questionnaire was administered to anatomy educators in higher level institutions in the ROI and UK. Inductive content analysis was used to identify the impact of UDL on student learning, engagement, and motivation, as perceived by the participants. The response rate was 23% (*n* = 61). Nineteen participants stated they knew of UDL. Of these, 15 had utilized UDL in their teaching of anatomy. Analysis indicated that the perception of UDL was mixed. However, the majority of responses relating to UDL were positive. The majority of the respondents were unaware of UDL but identified the frameworks' checkpoints within their curriculum, suggesting they have unknowingly incorporated elements of UDL in their curriculum design and delivery. There is a lack of information on the benefits of explicit utilization of UDL for engagement and motivation to learn anatomy in healthcare programs in the ROI and UK.

## 1 INTRODUCTION

Anatomy is an essential pillar of healthcare programs and the foundation for safe and effective practice (Sugand et al., 2010). Healthcare professionals have voiced their concern about poor anatomy knowledge among recent healthcare graduates (Bhangu et al., 2010; O' Keeffe et al., 2019) in particular their poor anatomical competency and lack of preparedness upon entering residency programs (Fillmore et al., 2016), suggesting that anatomy curriculum design requires reform. In the modern curriculum, there are constraints on the amount of time allocated to the formal teaching of anatomy within healthcare programs, and more specifically time dedicated to dissection (Harrison et al., 2019; Jeyakumar et al., 2020). In the Republic of Ireland (ROI) and United Kingdom (UK), anatomy is typically taught using an in‐person didactic format complemented with practical laboratory sessions to consolidate student learning (Smith et al., 2021). Both of these teaching modalities have been identified as critical for students' anatomy learning experience (Farkas et al., 2016). Recent literature indicates that these traditional methods should not be used in isolation but rather alongside new innovative methods to enhance accessibility of learning material and to promote student engagement and interest in anatomy (Dempsey et al., 2022; Iwanaga, Loukas, et al., 2021; Lochner et al., 2016). For example, studies have identified that modern teaching strategies, such as gamification, virtual and augmented reality which align with multiple means of representation, result in similar levels of knowledge acquisition and collaborative activity as traditional methods (Moro et al., 2021; Rezende et al., 2020). However, these modern methods have the added bonus of stimulating student autonomy and increasing satisfaction among learners (Alfalah et al., 2019; Rezende et al., 2020) all of which aligns with the Universal Design for Learning (UDL) framework. Furthermore, research has highlighted that certain teaching strategies may not suit all students, as students differ in academic preferences and abilities (Hu et al., 2018; Quinn et al., 2018; Ruthberg et al., 2020). The learning styles and social and cultural background of the student population is continually changing as more learners are traveling and migrating to enter third level education (Nortvedt et al., 2020; Vos et al., 2016). Specifically, in 2017 international students comprised 12.5% of the entire third level student population in the ROI (Department of Education, 2018) and this number is increasing as 18.4% of all graduates in the ROI in 2019 were international students (CSO, 2022). Additionally, widening participation agendas promote the accessibility of third‐level education (House of Commons, 2022). Thus, more students from diverse socio‐economic backgrounds or with learning difficulties or disabilities are entering higher education both in the ROI and UK (GOV, 2022; HEA, 2022). Therefore, to sustain student interest and engagement in anatomy education, educators need to adapt their curriculum to the present student population and educational environment (Chan et al., 2021; Jeyakumar et al., 2020; Kahu & Nelson, 2018).

In March 2020, all higher level institutions in the ROI and UK were required to pivot to online teaching during the global COVID‐19 pandemic (Franchi, 2020; Longhurst et al., 2020). Educators had to adapt quickly to ensure that students could continue to learn and engage with their material, albeit remotely (Evans et al., 2020). Anatomy educators became creative and innovative in their teaching methods (de Carvalho Filho et al., 2021; Dickinson & Gronseth, 2020; Evans et al., 2020; Harmon et al., 2021; Iwanaga, Loukas, et al., 2021; Yoo et al., 2021). For example, Harrell et al. (2021) incorporated online laboratory dissection videos, Flynn et al. (2021) introduced a 3‐dimensional anatomical modeling program to medical students, and Zarcone and Saverino (2022) used Leica Acquire, a virtual microanatomy application, to teach pathological anatomy. Additionally, new teaching methods such as Augmented Reality (AR) (Iwanaga, Terada, et al., 2021) and asynchronous and synchronous lectures (Byrnes et al., 2021; Harrell et al., 2021) were utilized. Opportunities for students to demonstrate their understanding in multiple formats such as presentations (Flynn et al., 2021; Keet et al., 2021) and online breakout rooms (Al‐Neklawy & Ismail, 2022) were also provided. As we emerge from the pandemic and return to on‐campus teaching there is an increasing awareness of the benefits of student engagement to allow students to express their knowledge and understanding in a format which is most appropriate and accessible for them (Singh et al., 2021). These are fundamental features of UDL, a learning theory first articulated in the United States (US) in 1990.

**FIGURE 1:**
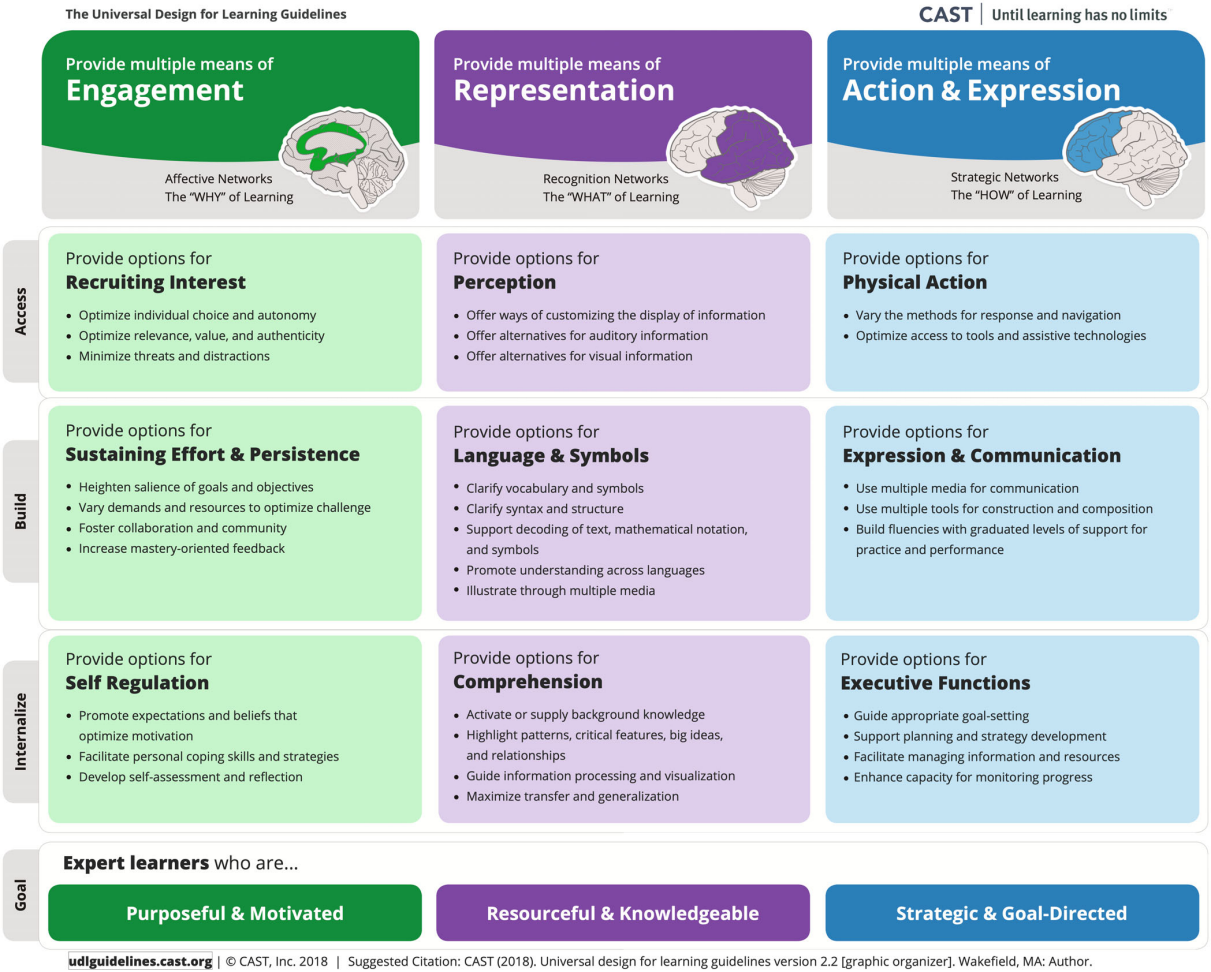
A schematic outlining the three Universal Design for Learning principles and their respective guidelines and checkpoints (CAST, 2021a)

Universal Design for Learning is an educational framework designed to ensure that all types of learners can participate, engage and thrive in the same learning environment (CAST, 2021a; Fornauf et al., 2021). The framework was developed by the Centre for Applied Special Technology (CAST) in the US as a guide for educators to design an accessible curriculum with the aim of changing the design of the learning environment rather than changing the learner (Bedrossian, 2018). The authors propose that anatomy students are encouraged to become life‐long and resilient learners through an enhanced educational experience that meets their learning preferences. To help accomplish this, three guiding principles were articulated, namely multiple means of engagement, multiple means of representation and multiple means of action and expression. Each principle is divided into three guidelines and each guideline has a set of associated checkpoints (31 in total) which educators may use in the design and delivery of curriculum, in order to enhance accessibility for all students (CAST, 2021a) (Figure 1). The aim of UDL is to afford the opportunity to all students to optimize their learning capabilities while also fulfilling the learning outcomes. If there are certain learning outcomes that need to be met or assessed, then there may be limited opportunity for flexibility. The authors contend that promoting and increasing knowledge and understanding of anatomy enhances safety in anatomy practice (Smith et al., 2016), which is a core component of the skillset needed for a professional degree (Lewis et al., 2016; Simons et al., 2022). However, the incorporation of UDL in the design and development of assessments in vocational degree programs requires further research as there is currently little information available. CAST advocate that providing learners with multiple options to engage with the learning material will help learners achieve their potential (CAST, 2021a).

Since the inception of UDL in primary school settings, educators in Canada and the US have reported that students retained increased amounts of class content, that student communication skills were enhanced and that participation in classroom activities had increased (Katz, 2015; Lowrey et al., 2017). A meta‐analysis carried out by Capp (2017) reported that UDL is an effective teaching strategy for enhancing the learning experience of all students with and without disabilities in environments ranging from second level to third level education (Capp, 2017). However, it must be determined if these promising results and benefits, as a result of embedding UDL in the curriculum of primary and secondary education, would translate to students enrolled in higher level institutions.

Black et al. (2015) reported that presenting learning material in various formats, regularly providing students with constructive feedback and assessing students using diverse methods was beneficial to learning in a cohort of students enrolled in a higher level institution, in the US, all of which aligns with UDL checkpoints (CAST, 2021a). Murphy et al. (2020) suggested that because UDL has been implemented in an effective manner to aid students with disabilities in their transition to higher level institutions, then perhaps it could potentially be used to help students without disabilities in their transition to higher level institutions (Murphy et al., 2020). The UDL framework incorporates strategies like flexibility and accessibility which any student enrolled in higher level education, regardless of ability, will find beneficial (Griful‐Freixenet et al., 2017).

The UDL educational framework offers potential for enhancing students' engagement in anatomy learning in higher level institutions (Dempsey et al., 2022). Many of the teaching strategies incorporated into anatomy education during the pandemic are aligned with the UDL framework, although none of the studies (Byrnes et al., 2021; Harmon et al., 2021; Harrell et al., 2021) specifically mention that UDL underpinned the change in approaches to teaching anatomy. Although there is a lack of published research about the explicit utilization of UDL in anatomy education in general (Dempsey et al., 2022), it is considered an effective tool to enhance student learning of anatomy with a recognized need for multiple means of engagement, representation, action and expression. Research by Balta et al. (2021) and Dempsey et al. (2022) shows that anatomy educators currently, and perhaps unknowingly, use various teaching methods and strategies when designing their curricula including the use of technology in its many forms (Bell et al., 2019; Ruthberg et al., 2020). Particularly, Ruthberg et al. (2020) incorporated mixed reality as an alternative to cadaveric dissection to utilize students' time more efficiently and, in turn, improve practical examination scores. They concluded that their mixed reality platform “HoloAnatomy” reduced the amount of time required for learning musculoskeletal anatomy without compromising student understanding, but they also acknowledged that not all students benefited from 3D resources and that individuals with less developed spatial abilities required more time viewing the 3D images (Ruthberg et al., 2020). This is an example of how implementing one teaching method in isolation will not cater for all students equally. Therefore, there is a need for a variety of teaching methods to ensure that content is accessible to all students. Bell et al. (2019) included the use of ultrasound in their curriculum to nurture an active learning environment for medical students studying the anatomy of the floor of the mouth, a teaching method which 97% of participating students (*n* = 31) agreed improved their learning. Furthermore, research has identified active learning as an essential element in creating a productive learning environment as it fosters student participation and engagement (Felder & Brent, 2009) all of which are aims of UDL (CAST, 2021a). The approach of Bell et al. (2019) to incorporate active learning to help students become independent thinkers, also aligns with the aim of UDL (CAST, 2021a). Universal Design for Learning caters for students with diverse learning needs, such as poor spatial recognition. This can be accomplished in anatomy curricula by including teaching strategies such as three‐dimensional visualization software (Jamil et al., 2019; Lone et al., 2021) which allows students to view the learning material in different ways, which in turn helps with comprehension among learners.

More specifically, recent publications have reported the use of UDL in healthcare programs. Dickinson and Gronseth (2020) described the benefits and utility of the UDL framework for surgical education during the COVID‐19 pandemic when face‐to‐face interaction was limited. Specifically, Dickinson and Gronseth (2020) used simulation, online resources, and mobile applications to aid the delivery of their curriculum to surgical residents in the US. Murphy et al. (2020) investigated, by means of an online survey, the implementation and knowledge of the tenets of UDL among occupational therapy educators in the US. They concluded that there is a need for more extensive use of UDL in preparation for teaching and continuing professional development as they propose its implementation would improve student learning and preparation for entering the discipline of occupational therapy (Murphy et al., 2020). Additionally, Murphy et al. (2020) highlighted throughout their study that, as the student population in higher level institutions and student preferences for learning are becoming more diverse, UDL will become the key to ensuring that all occupational therapy students can be ready for effective clinical practice. Studies analyzing healthcare education in higher level institutions indicate that when designing curricula, educators may incorporate methods which align with the UDL framework, without explicitly stating that UDL was utilized (Dempsey et al., 2022). Furthermore, anatomy educators may be utilizing the UDL framework but may not have measured or published the impact of its utilization in healthcare programs. For both reasons, an exploration of anatomy educators' knowledge and use of UDL is necessary and timely.

The aim of this study was to determine if anatomy educators in the ROI and UK were aware of the UDL framework and to assess if, and to what extent, they have been incorporating UDL in their curriculum design and delivery for healthcare students. Furthermore, the study explored if anatomy educators have identified or measured an impact on student learning, engagement, or motivation as a result of utilizing UDL; if there is an association between teaching experience and the incorporation of UDL; and finally, if educators have identified any barriers to implementing the UDL framework in their anatomy curricula.

## 2 MATERIALS AND METHODS

### 2.1 Study design

The authors developed and administered an anonymous online questionnaire to academic anatomy educators who were teaching in the anatomy departments of higher level institutions in the ROI and UK. A list of such higher level institutions was available on the Anatomical Society's website (Anatomical Society, 2021). The Anatomical Society seeks to promote and advance research and education in all aspects of anatomical science. The email addresses of anatomy educators from the above listed higher level institutions were accessed through each of the respective higher level institutions websites. The online questionnaire was distributed via email from the first author (A.M.K.D.) to potential participants. The questionnaire was accessible through the survey platform, Microsoft Forms (Microsoft Corp., Redmond, WA). Ethical approval was obtained from the Institutional Social Research Ethics Committee [Log 2021‐082].

### 2.2 Questionnaire design

The questionnaire was divided into two sections. Section one gathered demographic information such as academic position, methods of teaching and assessing anatomy and the healthcare programs on which educators taught anatomy. Section two focused on the respondents' knowledge, experience, and opinion of the UDL educational framework. The questions in section two were a mixture of both open and closed questions. Open‐ended questions were included to allow participants to elaborate and expand on questions. The questionnaire was open to participants for 12 weeks from July 5, 2021 to October 1, 2021. Reminders were sent out fortnightly via email to all potential participants. Although the questionnaire was distributed during the COVID‐19 pandemic, none of the questions specifically mention the pandemic.

### 2.3 Data analysis

All data were exported to Microsoft Excel (Microsoft Corp., Redmond, WA) and frequency tables were created for categorical variables. Data were entered manually into Statistical Package for Social Scientists (SPSS), version 22 (IBM Corp., Armonk, NY). Descriptive analyses were completed including the lambda coefficient to identify associations between teaching experience and the utilization of UDL. Inductive content analysis was used to explore anatomy educators' opinions of UDL through open‐ended questions (Elo & Kyngäs, 2008; Kyngäs, 2020). Verbatim quotes that captured the concepts articulated by a number of participants were included.

## 3 RESULTS

The overall response rate was 23% (*n* = 61). The majority of anatomy educators who participated in this study were at lecturer level (*n* = 29, 48%) and were located in England (*n* = 39, 64%). The teaching experience of the participants ranged from less than 1 year to over 20 years. The majority of anatomy educators who taught on undergraduate programs taught medical students. Similarly, the majority of anatomy educators who taught on graduate entry programs (to students who already had an undergraduate degree in another discipline) also taught medical students. A large variety of methods were utilized to teach anatomy. Plastic models were reported to be the most utilized method (*n* = 54, 89%), closely followed by imaging (*n* = 52, 85%), didactic teaching (*n* = 52, 85%), technology (*n* = 52, 85%) and prosections (*n* = 51, 84%). Teaching anatomy with microscopes was the least utilized method by anatomy educators (*n* = 16, 26%). Participating educators were asked to indicate the most commonly used assessment methods for anatomy. Multiple choice questionnaires (MCQ) were the most selected method of assessment among the respondents (*n* = 56, 92%), followed by short answer questions (*n* = 39, 64%). Student self‐assessment was the least utilized method of assessment (*n* = 15, 25%). All information from section one of the questionnaire is summarized in Table 1.

**TABLE 1:** Demographic information of participating anatomy educators

		Number of educators
Gender	Female	30 (49%)
	Male	30 (49%)
	Non‐Binary	1 (2%)
Academic Position	Professor	13 (21%)
	Senior Lecturer	15 (25%)
	Lecturer	29 (48%)
	Tutor	1 (2%)
	Other	3 (5%)
Location	Ireland	9 (15%)
	Northern Ireland	1 (2%)
	England	39 (64%)
	Scotland	9 (15%)
	Wales	3 (5%)
Teaching Experience	Less than 1 year	1 (2%)
	1–3 years	7 (11%)
	4–6 years	11 (18%)
	7–10 years	13 (21%)
	11–15 years	9 (15%)
	16–20 years	6 (10%)
	More than 20 years	14 (23%)
Undergraduate Programs	Dentistry	14 (23%)
	Diagnostic Radiography	2 (3%)
	Medicine	47 (77%)
	Nursing	7 (11%)
	Occupational Therapy	5 (8%)
	Physiotherapy	7 (11%)
	Speech and Language Therapy	8 (13%)
	Therapeutic Radiography	2 (3%)
Graduate Entry Programs	Dentistry	3 (5%)
	Diagnostic Radiography	1 (2%)
	Medicine	19 (31%)
	Physiotherapy	1 (2%)
	Speech and Language Therapy	2 (3%)
	Therapeutic Radiography	1 (2%)
Teaching Methods	Plastic Models	54 (89%)
	Didactic	52 (85%)
	Imaging	52 (85%)
	Technology	52 (85%)
	Prosections	51 (84%)
	Osteology	46 (75%)
	Surface Anatomy	46 (75%)
	Dissection	45 (74%)
	Gamification	28 (46%)
	Team‐based learning	28 (46%)
	Plastination	23 (38%)
	Kinesthetic	18 (30%)
	Peer‐instructed Learning	18 (30%)
	Microscopes	16 (26%)
Assessment Methods	Multiple Choice Questionnaires	56 (92%)
	Short answer Questions	39 (64%)
	Essay	27 (44%)
	Objective Structured Clinical Examination	22 (36%)
	Extended Match Questions	21 (34%)
	Oral Examination	20 (33%)
	Group based Assessment	16 (26%)
	Student Self‐assessment	15 (25%)

### 3.1 Awareness of UDL

Of the 61 respondents, only 19 (31%) stated that they had heard of the term UDL prior to completing the questionnaire. Of these 19 educators, 15 (79%) (and thus 25% of all respondents) had incorporated elements of UDL in their teaching of anatomy.

### 3.2 Incorporation of UDL

**FIGURE 2:**
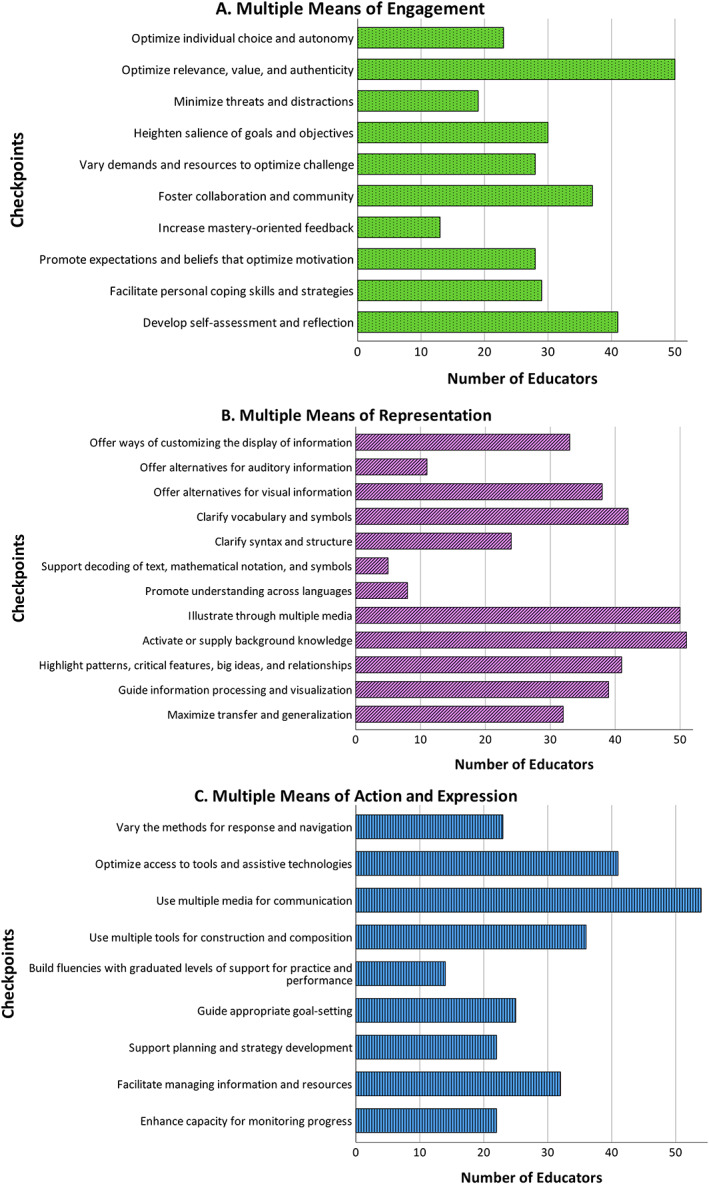
The number of anatomy educators whose anatomy curriculum aligned with each checkpoint of the (A) multiple means of engagement principle, (B) multiple means of representation principle, and (C) multiple means of action and expression principle

Respondents were asked whether the design and delivery of their anatomy curriculum aligned with any of the UDL checkpoints. Every respondent identified at least one checkpoint from each of the three UDL principles in their curriculum. The most utilized checkpoint, from the multiple means of engagement principle, was “optimize relevance, value and authenticity” (*n* = 50) and the least utilized was “increase mastery‐orientated feedback” (*n* = 13) (Figure 2A). The most utilized checkpoint from the multiple means of representation principle was “activate or supply background knowledge” (*n* = 51), followed closely by “illustrate through multiple media” (*n* = 50). The least utilized checkpoints from this principle were “support decoding of text, mathematical notation and symbols” (*n* = 5) and “promote understanding across languages” (*n* = 8) (Figure 2B). The most utilized checkpoint from the multiple means of action and expression principle was “use multiple media for communication” (*n* = 54) and the least utilized was “build fluencies with graduated levels of support for practice and performance” (*n* = 14) (Figure 2C).

Thirteen (21%) of the anatomy educators responded to the open‐ended question “If you have utilized elements of UDL, can you give an example of how?”. Eight of these 13 (62%) stated that they provide multiple means of representing the learning material. This is achieved by using several different formats including videos with closed captions, Microsoft PowerPoint slides with background information, digitally accessible documents, prosections, plastic models, and body painting. Five of these 13 (38%) respondents stated that they incorporate multiple means of engagement into their anatomy curricula by providing opportunities for the students to work together in pairs or small groups or by encouraging students to participate in self‐directed learning. Two of these educators (15%) provided multiple means of action and expression by integrating a variety of assessment methods into the curriculum and by supporting the students to present their learning in a manner which best suits their learning style. For example, they encouraged students to palpate and draw surface anatomy and incorporate anatomy maps or applications when presenting their learning to their peers.

### 3.3 Impact of UDL on student learning, engagement, or motivation

Inductive content analysis was used to identify the impact of UDL on student learning, engagement and motivation, as perceived by the participants. Of the 13 anatomy educators who responded to the open‐ended question “From your experience, how do you think UDL benefits teaching and learning within an anatomy module?”, the majority (*n* = 11, 85%) stated that UDL provides students with options for interacting with the learning material which in turn promotes engagement among students, empowers them to learn, creates a nurturing environment and makes it easier for students to access information. More specifically educators stated that UDL “*reduces the need for reasonable adjustment*,” “*enables different cognitive processes to process information giving a richer learning experience and leading to deeper knowledge and longer‐term retention*. *It offers flexibility and choice for learners but unless it is guided it can be overwhelming*,” and “*improves engagement*, *appeals to wider variety of learners*, *helps students with self‐assessment of progress and keeps instructors aware of individual learning needs of students*.” Furthermore, another educator commented that “*the (anatomical) language itself can be challenging and so it is important to ensure that this is clearly explained and providing a UDL approach gives students the best opportunity to be able to understand and engage not only with the physical anatomy but also with the language*” and “*variety is key as some students will engage with some aspects of the teaching and not in others*.”

Twelve respondents (20%) gave their opinion on whether the incorporation of UDL into curricula had an effect on student motivation to study anatomy. Two respondents did not think that the incorporation of UDL motivates students to learn anatomy. Three respondents said that they were not sure of the effect of UDL on motivation but that they were optimistic that it would have positive results. Specifically, one respondent said that they were “*not sure of the effect*” but that UDL has “*the potential for enhancing the student experience and in turn widening participation and motivation*.” The remaining seven educators (58%) agreed that UDL has an effect on student motivation. Four of these educators did not elaborate on whether it was a positive or negative effect but of the other three, one stated that they have seen an increase in motivation among graduate entry students, another said that “*it (UDL) gives them (students) more freedom to learn in a way that is suitable for them and allows them to explore what works for them*” and the final respondent said “*it (UDL) has made the subject more ‘fun’ and less dry*. *Anatomy is not something to be learned and forgotten*, *it is meant to be applied to healthcare*.”

**FIGURE 3:**
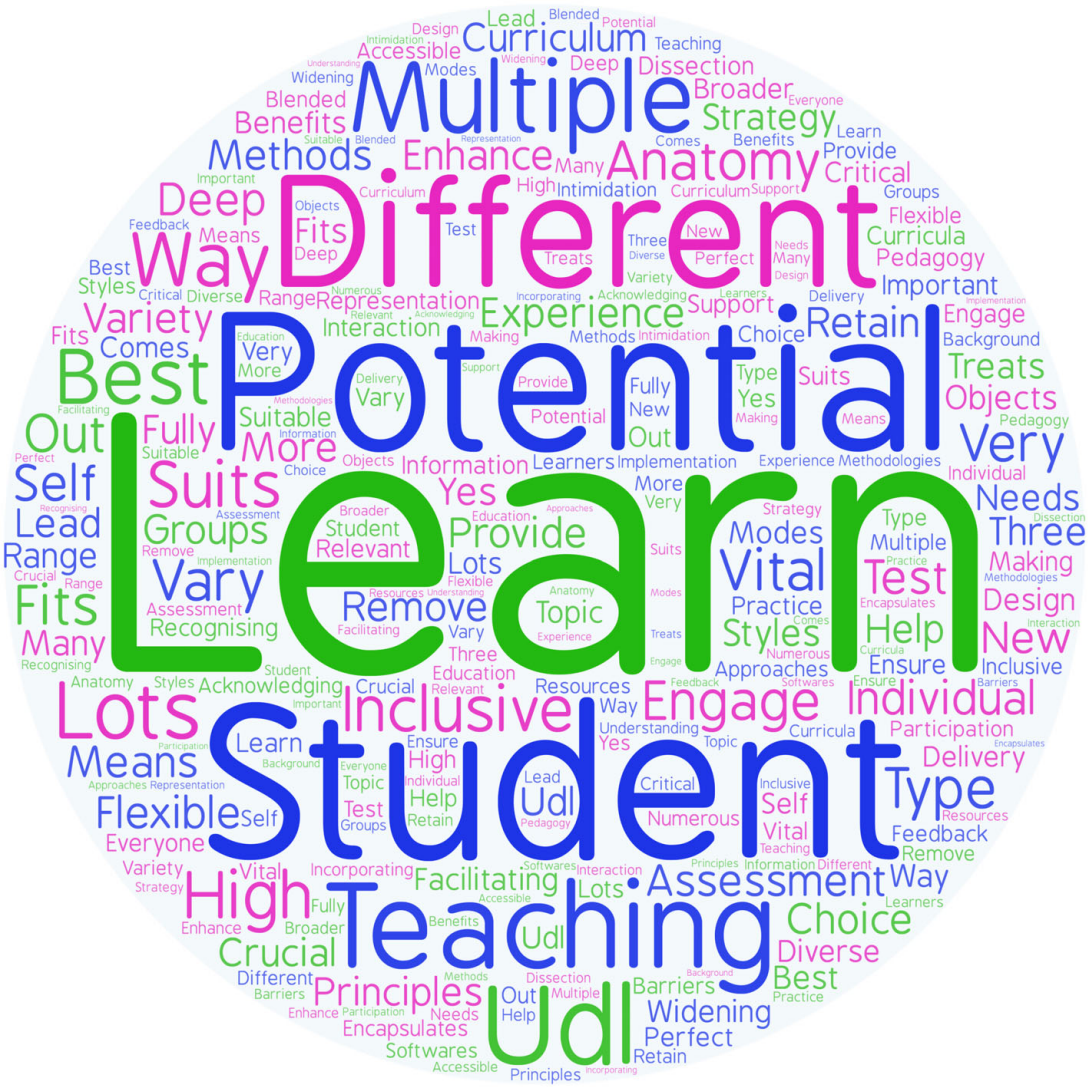
A word map created from anatomy educators' responses to the question “What potential, if any, do you see UDL having for teaching and learning within anatomy curricula?”

The anatomy educators were asked “if you are not familiar with UDL, from the description of the framework, what potential, if any, do you see UDL having for teaching and learning in anatomy curricula?”. Of the educators who were not familiar with UDL (69%), their opinion of the framework was mixed. Six (14%) of these respondents reported minimal or no potential of UDL application for teaching and learning in anatomy curricula. The remaining responses in relation to the potential of UDL were positive. For example, comments included “*lots of potential*, *especially for enhancing the student experience and being inclusive to different groups*,” “*potential to give students a learning experience that best suits their individual needs within an intended outcome framework*,” “*it would seem that UDL encapsulates those principles that are crucial for recognizing that individuals learn differently and facilitating such individual learning*,” and “*I think there is definitely scope for us to think more about providing variety of learning experiences within anatomy teaching*”. A word map was created to graphically represent the responses of participants to the open‐ended question “what potential, if any, do you see UDL having for teaching and learning within anatomy curricula?” to emphasize the recurring opinions. The size of a particular word in the figure corresponds with how frequently it appeared in the responses of the educators (Figure 3). Word maps align with the UDL framework under the multiple means of representation principle. In particular, they align with Guideline 1, Checkpoint 1.1 “Offer ways of customizing the display of information” (CAST, 2021a). For some readers, an image illustrating the most common responses may aid interpretation of the results.

### 3.4 Association between teaching experience and incorporation of UDL

A lambda coefficient statistical test was carried out to determine whether there was an association between respondents' teaching experience and their utilization of UDL. This resulted in a lambda score of 0.022 suggesting that there is no association between the number of years of teaching experience and the utilization of UDL among the responding anatomy educators.

### 3.5 Barriers to implementing the UDL framework in anatomy curricula

A small number (7%) of the anatomy educators commented on the practicalities of implementing UDL in anatomy curricula. Specifically, there was reference to a staff shortage “*with an intake of over 400 students per year and very few staff*, *personalization is not an option*” and “*it (UDL) is hard to formally implement when there is a staff shortage*.” Another educator said that UDL “*fits with best practice on teaching and learning but offers challenges in relation to assessment*.”

## 4 DISCUSSION

At the outset of the present study, the majority of the respondents were unaware of the UDL framework. However, once they gained some information about UDL and the associated principles and checkpoints, they realized that they had, seemingly unknowingly, been implementing elements of the framework in their curriculum design and delivery. This suggests a need to inform educators of UDL and the potential benefits for teaching and learning of anatomy to healthcare students, especially when many of the respondents recognized the potential benefits of UDL in their responses to the open‐ended questions. The present study did not investigate where educators, who were aware of the framework, had previously heard of UDL or if they received guidance on how to incorporate UDL into their curricula. Further research is needed to understand where and how educators are being made aware of the UDL framework, and whether supports are available to help guide them through the process of incorporating UDL into their anatomy curriculum design.

Fifteen of the respondents stated that they had been incorporating UDL into their anatomy curriculum and all 61 respondents identified at least one checkpoint from each of the UDL principles in their curriculum. This suggests that educators are already incorporating elements of the UDL framework, albeit potentially unknown to themselves. A scoping review analyzing the design and delivery of anatomy curricula in healthcare programs in higher level institutions revealed that when designing curricula, educators may incorporate methods which align with the UDL framework, without explicitly stating that UDL was utilized (Dempsey et al., 2022). However, there are still a number of checkpoints which very few respondents of this study identified in their curriculum design. For instance, only 21% of educators identified the checkpoint “increase mastery‐orientated feedback,” 31% identified the checkpoint “minimize threats and distractions” and 37% identified the checkpoint “optimize student choice and autonomy” (Figure 2A). Each of these checkpoints is categorized under the multiple means of engagement principle which helps foster self‐directed and motivated learning. Various studies have shown that providing students with autonomy over their own learning is vital to sustain engagement and interest in the subject matter (Alsharari & Alshurideh, 2021; Goodman et al., 2021; Hensley et al., 2021). Similarly, it has been shown that providing students with immediate feedback allows students to engage with the learning process (Blondeel et al., 2022; Young et al., 2020). There is no mandatory number of UDL checkpoints which educators should implement (Rao et al., 2014), as some checkpoints are not appropriate for all subjects. For example, under the multiple means of representation principle, the checkpoint “support decoding of text, mathematical notation and symbols” is difficult to implement within anatomy curricula and therefore it was not surprising that only 8% of the respondents identified this checkpoint in their own curriculum design. Similarly, few respondents (13%) identified the checkpoint “promote understanding across languages” (Figure 2B). In comparison, a high number of respondents stated that their curriculum aligned with checkpoints such as “activate or supply background knowledge” (84%), “illustrate through multiple media” (82%) and “highlight patterns, critical features, big ideas and relationships” (67%) (Figure 2B), all of which are from the multiple means of representation principle and have been established as influencing successful student learning across various disciplines (Manthra Prathoshni et al., 2018; Vieira et al., 2019; List et al., 2021; Ulfa et al., 2021). Specifically in anatomy education, Zafar and Zacher (2020) concluded that representing anatomy material in various formats, such as complementing the use of cadavers with AR, increased enjoyment and engagement among dental students. In relation to the multiple means of action and expression principle, some of the least identified checkpoints were “support planning and strategy development” (38%), “enhance capacity for monitoring progress” (36%) and “vary the methods for response and navigation” (36%) (Figure 2C). However, studies have identified that varying the way students navigate and participate in the learning environment, and guiding student goal‐setting helps nurture strategic and goal‐directed anatomy learners (Donkin & Rasmussen, 2021; Eleazer & Scopa Kelso, 2018; Grønlien et al., 2021; Hernandez et al., 2020). Incorporating each of these guidelines would provide learners with the opportunity to express their knowledge in a manner which is most appropriate to them, and to track their progress, which in turn would allow them to identify areas where they may be struggling, or indeed excelling, all of which helps learners to become strategic and goal driven (CAST, 2021a). Edyburn and Edyburn (2021) describe the essential practices required to help any educator implement UDL in a manner which provides meaningful support for the diverse student population so that every learner can be successful. The authors emphasize that there is a requirement to understand the philosophy of UDL, but additionally that there is a need to bridge the gap between knowing about and implementing UDL. The authors propose the utilization of UDL for the design and delivery of anatomy curricula, as UDL encapsulates a number of pedagogical theories, such as self‐determination theory (SDT) (Deci & Ryan, 2000; Hu & Zhang, 2017), generative learning theory (GLT) (Brod, 2021; Wittrock, 1974) and cognitive flexibility theory (CFT) (Spiro et al., 1991) into one framework. Universal Design for Learning also aligns with the FAIR principles postulated by Harden and Laidlaw (2016). Therefore, educators may look to one framework for guidance, when designing their curriculum delivery, rather than multiple different theories.

The present study illustrates the knowledge and perceived impact of UDL among anatomy educators in the ROI and UK. At the outset of this study, many of the respondents stated that they were not aware of the UDL framework, but at the end of the questionnaire they stated that they believed they were implementing elements of the framework in their curriculum design and delivery. These anatomy educators utilized various accessible and inclusive teaching methods and, in a number of cases, noticed improvements in student motivation and engagement as a result. The questionnaire provided educators with a very basic description of UDL. Perhaps with more detailed information they would be able to identify the areas in their curriculum that could be updated or modified. Teaching methods that align with the UDL framework have been reported to have a positive impact on learning among students with a variety of learning preferences across an array of programs in a higher level institution in the US (Black et al., 2015). The authors concluded that representing learning material in various formats, regularly providing students with constructive feedback, and assessing students using diverse methods was beneficial to student learning, all of which aligns with UDL checkpoints (CAST, 2021a). Murphy et al. (2020) suggested that since UDL has been effectively implemented in aiding the transition of students with disabilities to higher level institutions, then perhaps it could potentially be used to help students without disabilities in their transition to higher level institutions (Murphy et al., 2020). Furthermore, the UDL framework incorporates strategies like flexibility and accessibility to learning material which any higher level education student, regardless of ability would find beneficial (Griful‐Freixenet et al., 2017).

The respondents' teaching experience ranged from less than 1 year to more than 20 years. There was no association between experience and the utilization of UDL, indicating that the educators' awareness of UDL and its implementation in anatomy curricula is not reliant on the extent of teaching experience.

Inductive content analysis was used to explore the participating anatomy educators' opinions of UDL. From this analysis it became clear that, according to the respondents, the main barrier of incorporating the UDL principles into anatomy curricula is a staff shortage. Staff shortage in anatomy programs has been documented (Kramer et al., 2020; Wilson et al., 2020). Thus, there may be a negative association with implementation of a new teaching framework to anatomy curricula due to the increased workload that typically accompanies curricular change. However, respondents may not yet be aware of recent publications guiding educators to incorporate UDL into their curriculum design and delivery (Cotán et al., 2021; Edyburn, 2021; Luke, 2021; Xie & Rice, 2021). Arguably, once anatomy educators are more knowledgeable about UDL, they will have an increased understanding of how easily they can tailor their curricula to accommodate all students, without the major workload they may have originally thought was required. Lee and Griffin (2021) highlight that not all educators will be comfortable implementing UDL in their curriculum design and delivery right away. Rather, they will require time to become confident with potentially new teaching strategies. Incorporation of UDL into assessment is highlighted as a barrier by one respondent who stated that UDL “*fits with best practice on teaching and learning but offers challenges in relation to assessment*.” This could be addressed by the suggestions compiled by CAST to help educators implement UDL strategies in the design and delivery of their assessments. They propose that educators consider which actions are relevant to the information being assessed and which actions can be supported or varied in order to obtain an accurate account of what each individual has learned (CAST, 2021b). The authors propose including a variety of question styles within an end‐of‐year assessment paper such as multiple choice questions (MCQs), extended match questions, short answer questions, labelling of diagrams, true or false questions, and essays. For continuous assessment, educators could allow the students to decide how they want to demonstrate their knowledge, either through essay, PowerPoint presentation, or pre‐recorded video, while adhering to the learning outcomes of the module. Providing all the students with the same options removes the perception of unfairness, as they all have the same opportunity to choose which format they prefer to be examined in.

Specific examples of how anatomy educators may incorporate UDL into the design and delivery of their anatomy curricula include: highlight the relevance of the information, the clinical significance, if there are any notable anatomical variations; vary the level of demands posed to students, include both straight forward, simple questions and higher order questions. Incorporation of these examples of UDL would thus serve to challenge the high performing students to sustain engagement, while also catering for the weaker student so that they do not become overwhelmed and discouraged. Other suggestions of how UDL could be incorporated into anatomy education include: provide immediate feedback via self‐assessed practice examinations or using game‐based learning platforms like Kahoot! During lectures or tutorials; display information in a variety of formats including text, images, video, animations and voice over narration; allow students to choose, when appropriate, how to complete an assignment using PowerPoint presentation, video presentation or written essay.

The COVID‐19 pandemic provided a unique opportunity for educators in higher level institutions to implement UDL more extensively in anatomy curricula, as educators had to adjust and adapt their curriculum design and delivery rapidly. The multiple ways in which anatomy can be taught both successfully and inclusively were highlighted when educators were forced to teach anatomy remotely (Byrnes et al., 2021; Goldman et al., 2021; Patra et al., 2021). Longhurst et al. (2020) investigated the adaptations made specifically to anatomy education in the ROI and UK in response to the COVID‐19 pandemic. It was reported that the most commonly expressed concern among anatomy educators from the participating higher level institutions (*n* = 14) was in relation to the time investment required to develop new resources to replace traditional lectures and practical classes. This concern overlaps with the concerns expressed by the anatomy educators participating in this study with regards to incorporating UDL into their curriculum design. Furthermore, 36% of the respondents in the Longhurst et al. (2020) study highlighted that there was a reduction in student engagement since the start of the pandemic. Engagement is one of the main three principles of the UDL framework (CAST, 2021a). In Australia and New Zealand, Pather et al. (2020) concluded that flexibility and adaptability were essential for the continuity of anatomy education programs during the COVID 19 pandemic, both of which are aims of UDL. Perhaps if educators were more aware of UDL and the ways in which it can be incorporated into curriculum design and delivery, it may have eased some of the burden and stress among educators, in relation to teaching, during the COVID‐19 pandemic.

The study is not without limitations. There was a 23% response rate and therefore the results cannot claim to be representative of all anatomy educators in ROI and the UK. However, the demographic of the respondents was quite varied, thus removing an opportunity for bias. Furthermore, only educators whose email address was accessible through their institutions website were contacted to participate. Although this study was carried out during the COVID‐19 pandemic, the questionnaire did not specifically mention the pandemic. Therefore, it cannot be stated for certain whether the teaching strategies utilized by the respondents were always being used, or whether they were new strategies utilized in response to a global pandemic.

In conclusion, this study highlights that anatomy educators from higher level institutions in the ROI and UK are implementing teaching strategies which align with the UDL framework. However, the majority of respondents were not aware that a specific name can be used to collectively identify the teaching methods used. There is still a lack of information on the benefits of the explicit utilization of UDL in anatomy curricula of healthcare programs in higher level institutions. Furthermore, the authors conclude that it would be beneficial to introduce the UDL framework to anatomy educators in the ROI and UK. The potential positive impact of the explicit utilization of UDL on healthcare students' learning, engagement, motivation, and experience in higher level education is evident. The optimal method of distributing this information to educators requires consideration and research.
